# Static all-atom energetic mappings of the SARS-Cov-2 spike protein and dynamic stability analysis of “Up” versus “Down” protomer states

**DOI:** 10.1371/journal.pone.0241168

**Published:** 2020-11-10

**Authors:** Michael H. Peters, Oscar Bastidas, Daniel S. Kokron, Christopher E. Henze

**Affiliations:** 1 Department of Chemical and Life Science Engineering, Virginia Commonwealth University, Richmond, Virginia, United States of America; 2 College of Biological Sciences, University of Minnesota, Minneapolis, Minnesota, United States of America; 3 NASA Ames Research Center, Moffett Field, California, United Sates of America; CIC bioGUNE, SPAIN

## Abstract

The SARS-CoV-2 virion responsible for the current world-wide pandemic COVID-19 has a characteristic Spike protein (S) on its surface that embellishes both a prefusion state and fusion state. The prefusion Spike protein (S) is a large trimeric protein where each protomer may be in a so-called Up state or Down state, depending on the configuration of its receptor binding domain (RBD) within its distal, prefusion S1 domain. The Up state is believed to allow binding of the virion to ACE-2 receptors on human epithelial cells, whereas the Down state is believed to be relatively inactive or reduced in its binding behavior. We have performed detailed all-atom, dominant energy landscape mappings for noncovalent interactions (charge, partial charge, and van der Waals) of the SARS-CoV-2 Spike protein in its static prefusion state based on two recent and independent experimental structure publications. We included both interchain interactions and intrachain (domain) interactions in our mappings in order to determine any telling differences (different so-called “glue” points) between residues in the Up and Down state protomers. The S2 proximal, fusion domain demonstrated no appreciable energetic differences between Up and Down protomers, including interchain as well as each protomer’s intrachain, S1-S2 interactions. However, the S1 domain interactions *across* neighboring protomers, which include the RBD-NTD cross chain interactions, showed significant energetic differences between Up-Down and Down-Down neighboring protomers. This included, for example, a key RBD residue ARG357 in the Up-Down interaction and a three residue sequence ALA520-PRO521-ALA522, associated with a turn structure in the RBD of the Up state protomer, acting as a stabilizing interaction with the NTD of its neighbor protomer. Additionally, our *intra* chain dominant energy mappings within each protomer, identified a significant “glue” point or possible “latch” for the Down state protomer between the S1 subdomain, SD1, and the RBD domain of the same protomer that was completely missing in the Up state protomer analysis. Ironically, this dominant energetic interaction in the Down state protomer involved the backbone atoms of the same three residue sequence ALA520-PRO521-ALA522 of the RBD with the amino acid R-group of GLN564 in the SD1 domain. Thus, this same three residue sequence acts as a stabilizer of the RBD in the Up conformation through its interactions with its neighboring NTD chain and a kind of latch in the Down state conformation through its interactions with its own SD1 domain. The dominant interaction energy residues identified here are also conserved across reported variations of SARS-CoV-2, as well as the closely related virions SARS-Cov and the bat corona virus RatG13. We conducted preliminary molecular dynamics simulations across 0.1 *μ* seconds to see if this latch provided structural stability and indeed found that a single point mutation (Q564G) resulted in the latch releasing transforming the protomer from the Down to the Up state conformation. Full trimeric Spike protein studies of the same mutation across all protomers, however, did not exhibit latch release demonstrating the critical importance of interchain interactions across the S1 domain, including RBD-NTD neighboring chain interactions. Therapies aimed at disrupting these noncovalent interactions could be a viable route for the physico-chemical mitigation of this deadly virion.

## Introduction

SARS-Cov-2 is a positive-sense RNA virus responsible for the disease COVID-19 which was recently declared a world-wide pandemic by the WHO. SARS-CoV-2 virion enters human epithelial cells by attachment of it’s prefusion-form Spike protein (S) with a specific cell surface receptor ACE2 (Angiotensin Converting Enzyme 2) [1–3]. The Spike protein is a large, trimeric protein (1273 residues per chain) whose Receptor Binding Domain (RBD) undergoes somewhat unusual large dynamic transformations reflecting the overall flexibility of the S1 domain of the Spike Protein. In general, the S1 domain represents a prefusion domain (residues 27-677) and the S2 domain (residues 681-1273) is the fusion domain. In general, the S2 or fusion machinery domain of S is relatively rigid with strong noncovalent interactions facilitated by helical secondary structures, whereas the S1 domain, which contains the RBD and N-terminal domain (NTD), is flexible and characterized by beta strand structural motifs. The S1 domain consists of subdomains NTD (N-terminal domain), the RBD (Receptor binding domain; residues 319-527), and S1 subdomains (SD1) and (SD2) that are the most proximal to the S2 domain [1–3]. We note that the S1 domain of the Spike protein is shed in the transition from the prefusion state to the fusion state of this virion; those transformational aspects are not considered here and our focus is on the prefusion state. In the so-called “Up-state” of the RBD, the (prefusion) protein is able to bind to ACE2 (Angiotensin Converting Enzyme 2) and infect (via a transformation to its fusion state) human epithelial cells (Type I and II pneumocytes; also, alveolar macrophage and nasal mucosal cells), but in the “Down-state” of the RBD the Spike protein is believed to be inactive to ACE2 binding and to cellular infection.

The specific structural details of the RBD in the Up versus Down states in SARS-CoV-2 have recently been elaborated and compared across the S proteins of *β* corona viruses [4] and Up versus Down states have been reported [5] for SARS-CoV and MERS-CoV. Li et al. [6], based on Cryo-EM studies of recombinant Spike protein structures, suggest an earlier prefusion conformation prior to the RBD-Down state. In general, however, the exact origins of these configurations in trimerically folded states and their stability and possible transitions remain largely unanswered. For example, it is still not clear whether these states exist simply randomly, are folded states from their nascent or some kind of pre-folded structures, or are obtained by post-folded modifications orchestrated by the virion or its environment. So, the detailed study of the key binding residues and dynamics within this complex protein in its different conformations may shed light into the stability and possible transitions between the two states. Recently unpublished long time Molecular Dynamics (MD) studies of an isolated Spike Protein by the Shaw Group [7] noted that the protomers tended to persist in their initial states, i.e, Down states remain Down and Up states remain Up. Wrapp et al. [1] recently determined the molecular structure of the S protein ectodomain trimer from SARS-Cov-2 by cryo EM with one protomer Up and two protomers Down (PDB ID 6VSB). Additionally, Walls et al. [2], also recently and independently determined the S protein molecular structure of SARS-CoV-2 using Cryo EM with one published structure also given in the one Up and two Down state (PDB ID 6VYB) and another published structure in the three Down state (PDB ID 6XXX). Cai et al [8] also determined the pre- and post-fusional structure of the full length S protein to 2.9A and 3.0 A, respectively, using Cryo EM. Interestingly, no studies have shown more than one Up state protomer within any wild-type S trimer, and this may point to the possibility of interactions between neighboring chains as a critically important stabilizing factor for the S ectodomain in its prefusion conformation.

In order to better understand the differences between the Up and Down protomer states and the possible transitions between them, we conducted an all-atom interacting energy landscape mapping of the entire Spike protein from their *.pdb (Protein Data Bank) structure files (6VSB and 6VYB), where one of the protomers is in the Up state and two are in the Down state. This allows us to identify key interaction energy “glue” points (both interchain and intrachain) associated with relatively strong non-covalent atom-atom interactions between residues that may be responsible for specific persistent domains of this complex trimeric protein. In doing so, we are able to identify some unique and critically different glue point residues between the Up and Down protomers within the overall trimeric structure. This analysis was then used to guide molecular dynamic studies incorporating single point mutations of the S protein in order to investigate the local dynamic stability between the Up and Down states. We note that these key residues will be shown to be conserved across the closely related virions SARS-CoV and the bat corona virus RatG13, as well as known variations of the novel corona virus. Comparative analyses between Up and Down state protomers, such as those given here, may provide insights useful for vaccine development in COVID-19, for example via multiple and single point mutations to favor the Up conformational state [4], potentially new therapeutic targets aimed at maintaining the Down state of the Spike protein [9], or dismantling of the entire trimeric prefusion structure by the disruption of key interchain interactions given here.

## Materials and methods

### All-atom energetic mappings

The SARS-CoV-2 Spike protein structures considered here consist of three chains or protomers (A, B, and C chains) of which one chain is given in the so-called “Up” state of its RBD and the remaining two chains are in their “Down” state. We energetically mapped the interchain interactions “Up-Down” and “Down-Down” and specific domain interactions (*intra*chain interactions) for the Up and Down state protomers, including S1 and S2 domain interactions and sub domains of S1 that include the RBD domain.

Following our recently published study on *Aβ*42 amyloid fibrils [10], we analyzed the recently published trimeric structures of SARS-CoV-2 Spike protein (S) (PDB ID’s: 6VSB and 6VYB) according to the Coulombic (charge and partial atomic charge) and Lennard-Jones (Born and van der Waals forces) atom-atom interaction forces as laid out in the open-source energy mapping algorithm developed by Krall et al [11]. This mapping algorithm efficiently parses the strongest non-covalent atom-atom interactions and their inter-atomic distances from structure file data according to empirically established criteria based on the *AMBER*03 force field model to ensure that all dominant interactions are accounted for. These energetic mappings, for example, allowed us to discern important structural differences leading to greater adhesive strengths of *Aβ*42 oligomers versus their *Aβ*40 counterparts. Following our previous studies, the parsing criteria were taken as the upper limit of −0.1*kT* units for Lennard-Jones (van der Waals) criteria and −0.3*kT* units for Coulombic interactions, although lower values can also be specified in the analysis part of the mappings in order to further refine the results [11].

### Molecular dynamics

Explicit solvent molecular dynamics (MD) simulations of the novel coronavirus Spike protein were performed using the NAMD2 program [12]. We used the CHARMM-Gui [13] with the CHARMM36m force field along with TIP3P water molecules to explicitly solvate the proteins and add any missing residues from the experimental structure files. Disulfide bonds and glycosylated sites were all included. Simulations were carried out maintaining the number of simulated particles, pressure and temperature (the NPT ensemble) constant with the Langevin piston method specifically used to maintain a constant pressure of 1 atm. We employed periodic boundary conditions for a water box simulation volume as well as the particle mesh Ewald (PME) method with a 20 Å cutoff distance between the simulated protein and water box edge. The integration time step was 2 femtoseconds with our protein simulations conducted under physiological conditions (37 C, pH of 7.4, physiological ionic strength with NaCl ions, LYS and ARG were protonated and HIS was not).

## Results and discussion

### *Inter*chain interactions

**Fig 1:**
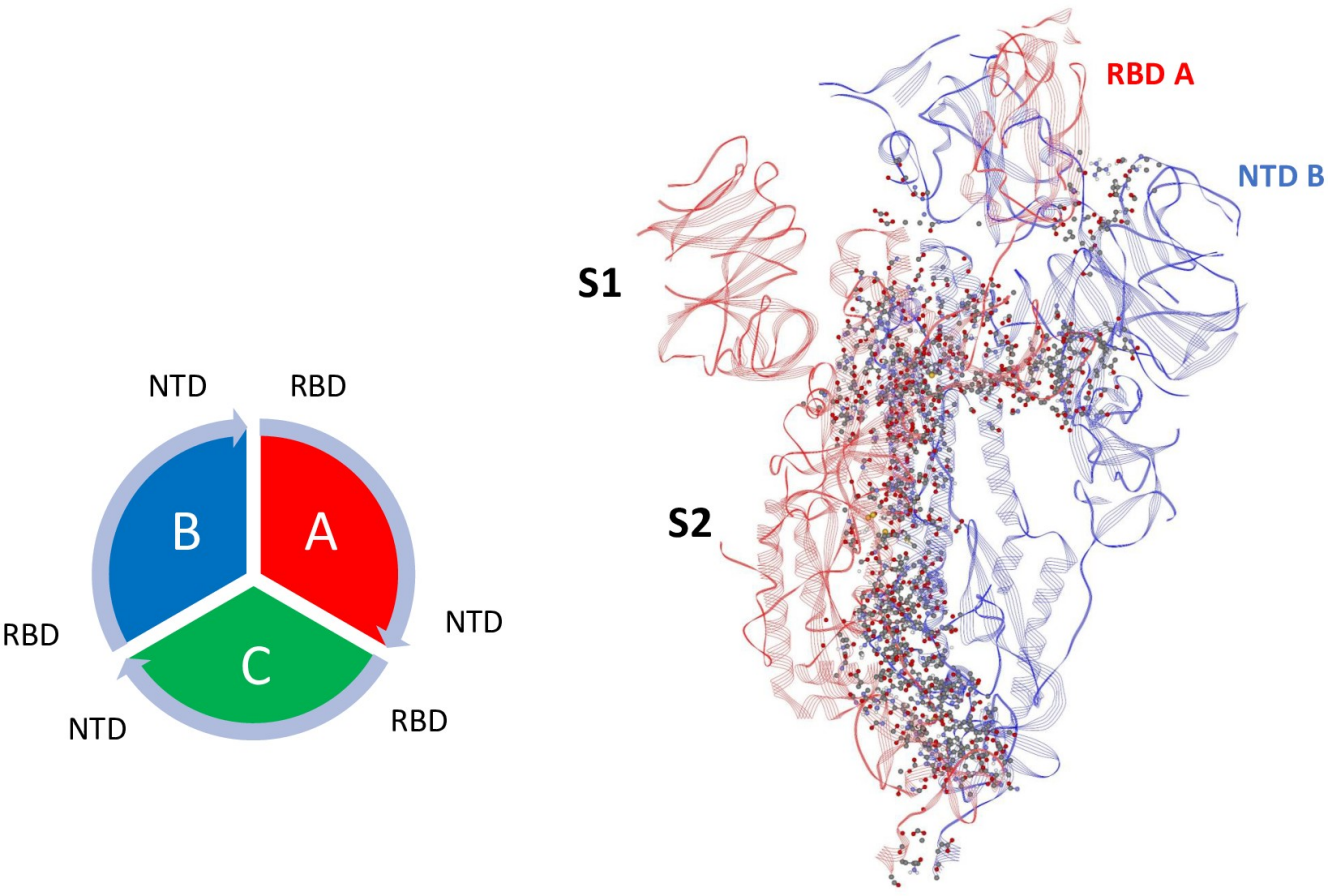
Dominant energy landscape for the interaction of chain A (Up; red) with chain B (Down; blue) for 6VSB. The dominant atom-atom interactions are shown by ball and sticks. Also shown is the overall chain interaction configuration looking at the trimer from the top view.

**Fig 2:**
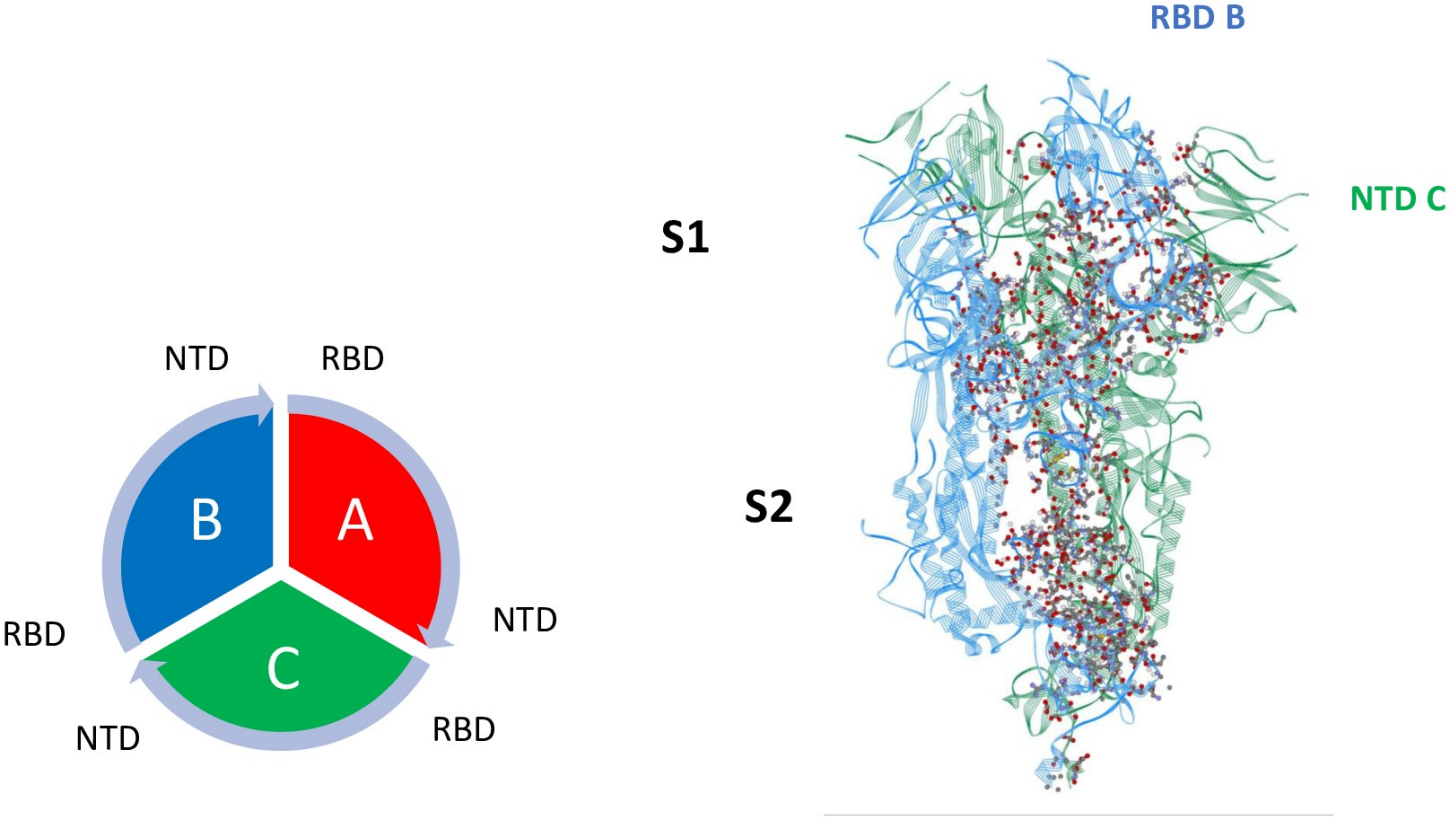
Dominant energy landscape for the interaction of chain B (Down; blue) with chain C (Down; green) for 6VSB. The dominant atom-atom interactions are shown by ball and sticks.

Mappings of the dominant atom-atom interactions among the residues for the Up-Down (A-B) and Down-Down (B-C) states are illustrated in Figs 1 and 2, respectively, for 6VSB (S1 and S2 Tables in S1 File). As expected, the majority of the dominant interactions are within the S2 domain, and the entire three chain structure is greatly stabilized by this feature. There are a number of stabilizing interactions involving the SD1/SD2 domain (residues 533-677) of any protomer with the S2 and NTD domain of neighboring protomers (S1, S2 Figs in S1 File). The pattern of the S1-S1 interchain interactions involving the RBD of any chain to the N-terminal domain (NTD) of its adjacent neighbor is potentially important as shown in Figs 1 and 2 for clarity. Similar results were obtained for the 6VYB structure (S3 and S4 Tables in S1 File).

**Fig 3:**
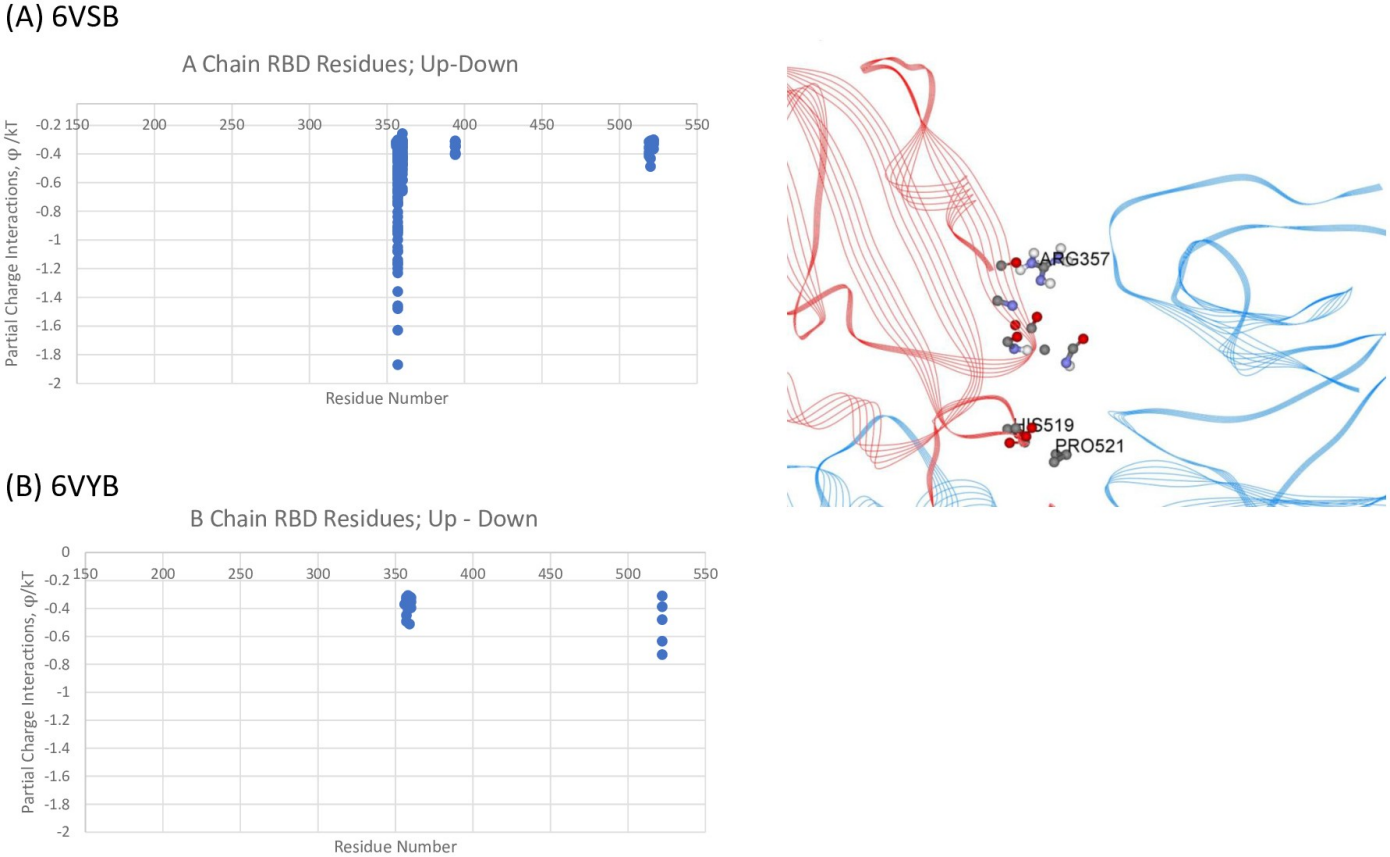
(A) Dominant Energy Landscape for the Interaction of the RBD Chain A (Up;red) with NTD Chain B (Down;blue) for 6VSB; oblique, top view. Only A Chain RBD residue/atoms are shown. (B) Mappings for Up-Down 6YVB. See S1–S10 Tables in S1 File for all data.

**Fig 4:**
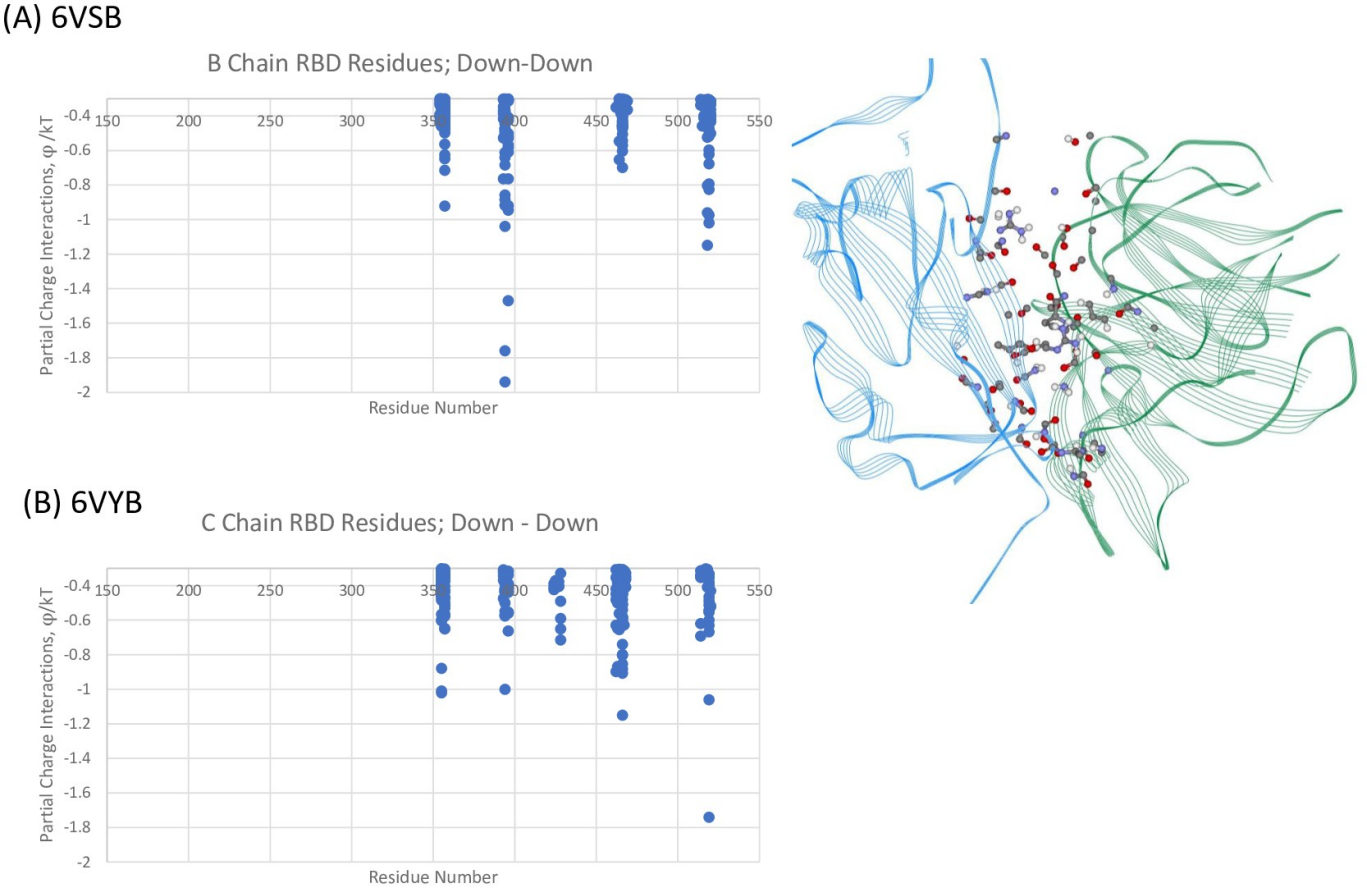
Dominant energy landscape for the interaction of the RBD of chain B (Down;blue) with the NTD of chain C (Down;green) for 6VSB; oblique, top view. Only B Chain residue/atoms are shown. (B) Mappings for Down-Down 6VYB. See S1 File.

We specifically examined the interchain interactions between the RBD with the NTD of its neighbor protomer for both the Up-Down (A-B) and Down-Down (B-C) interactions for 6VSB and 6VYB, shown in Figs 3 and 4, respectively. As seen, there is very good agreement of the specific dominant energetic interactions predicted across the two independently determined structure files. For the Up-Down, RBD-NTD interactions, RBD residues ARG357 and ALA520-PRO-521-ALA522 play key stabilizing roles. The differences in magnitudes shown in Fig 3 across the two structures are associated with a slightly more distal location of the Up chain for 6VYB as compared to 6VSB, but the residues are the same in either case. For the Down-Down RBD-NTD interactions, additional RBD residues ASN394, TYR396, and ARG466 on the average tend to strengthen the binding of these adjacent subunits.

### *Intra*chain interactions

We next looked in detail at *intra*chain interactions for both the Up and Down protomers in order to attempt to discern any telling differences in these two states.

#### S1-S2 intrachain interactions

We first energetically mapped the S1 domain (residues 1-677; not including missing residues) to the S2 domain (residues 682-1273; not including missing residues) of both the Up protomer and Down protomer separately, as shown in S5 and S6 Tables in S1 File. Overall, we found no discernable differences in the energetic interactions between the Up and Down states, which again may not be surprising considering the RBD of S1 (residues 319-527) is distal to the S2 domain whether in the Up or Down protomer state. Additionally, the proximal S1 subdomains near the S1-S2 border, SD1/SD2 domains (residues 533-677), also showed no discernable differences between the Up and Down states in it’s interaction with S2. We, therefore, next compared the SD1/SD2 domains to RBD domain interactions within each protomer in the Up or Down state, since these states have clear differences in separation distances owing to the more distal RBD in the Up state.

#### SD1/SD2-RBD intrachain interactions

**Fig 5:**
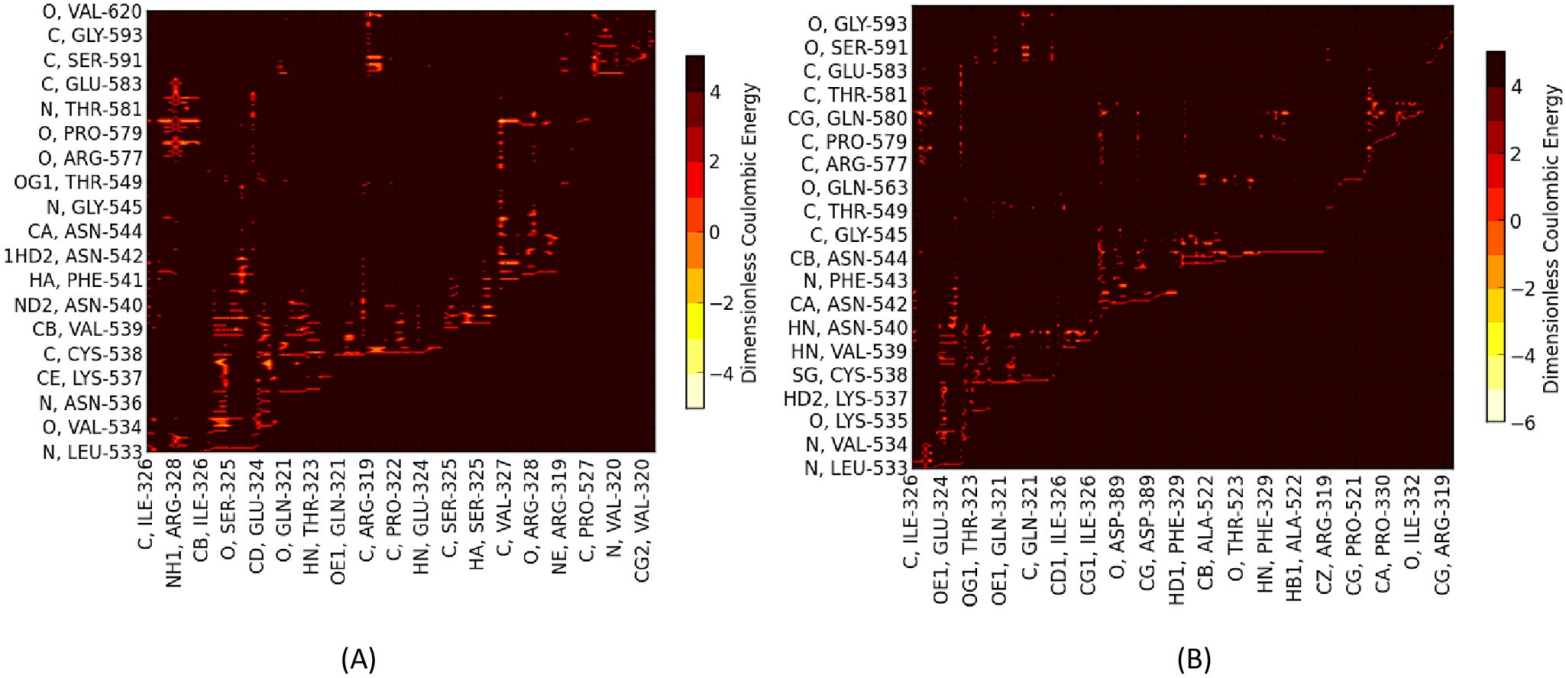
(A) Dominant Energy Landscape for Partial Charge Interactions between SD1/SD2 residues of Chain A (Up) (vertical axis) and RBD of Chain A (Up) (horizontal axis) (PDB ID: 6VSB). (B) Dominant Energy Landscape for Partial Charge Interactions between SD1/SD2 and RBD of Chain B (Down) (6VSB).

**Fig 6:**
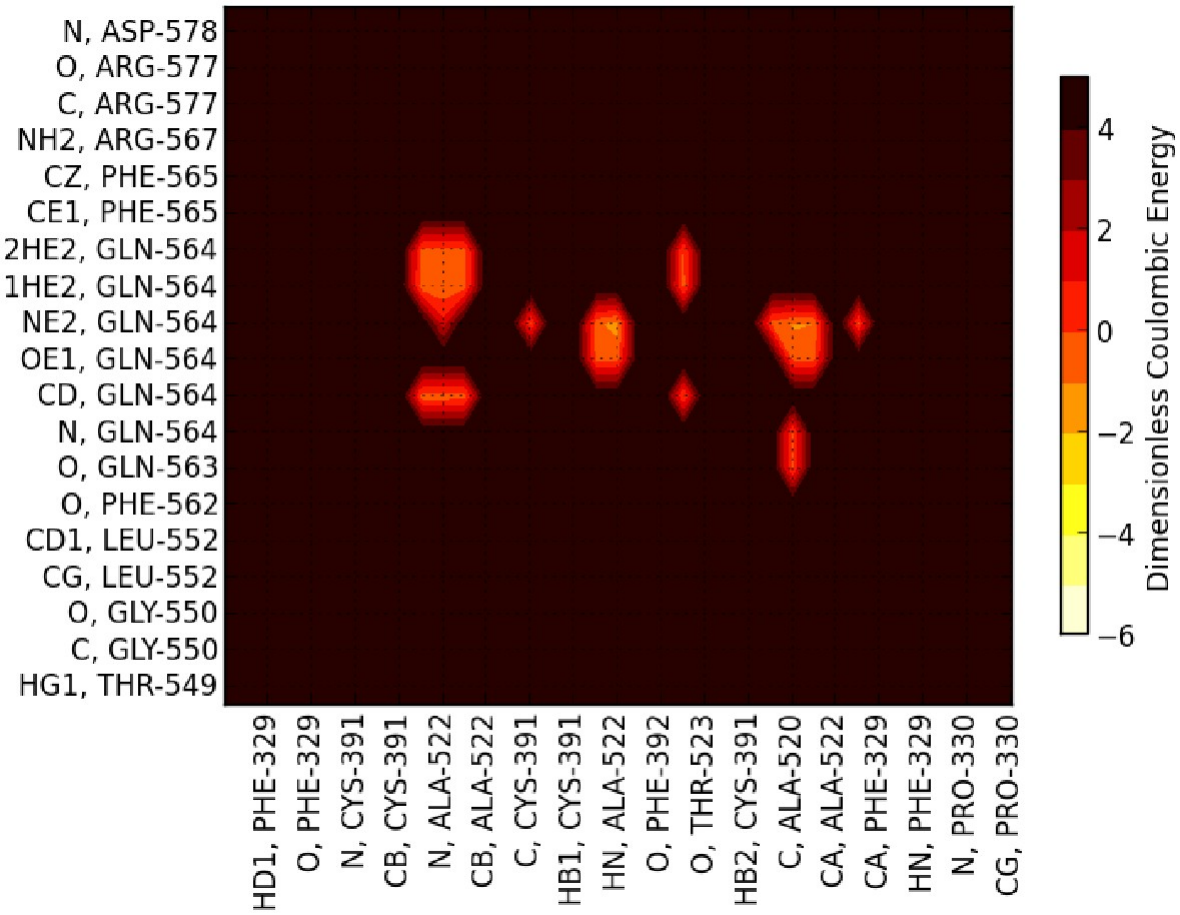
Zoomed dominant energy landscape for partial charge interactions between SD1/SD2 residues (vertical axis) and RBD residues of chain B (horizontal axis)showing the conspicuously unique residue interactions. Note that dominant van der Waals interactions involving PRO 521 backbone atoms are not shown here; see S7 and S8 Tables in S1 File.

Overall the energetic mappings between Up and Down states for these two S1 domains (RBD-SD1/SD2) appear very similar for any structure, as shown, for example, by the dominant partial charge interactions (Fig 5; S7 and S8 Tables in S1 File. for 6VSB) with one important difference as shown in the zoom illustration of Fig 6.

**Fig 7:**
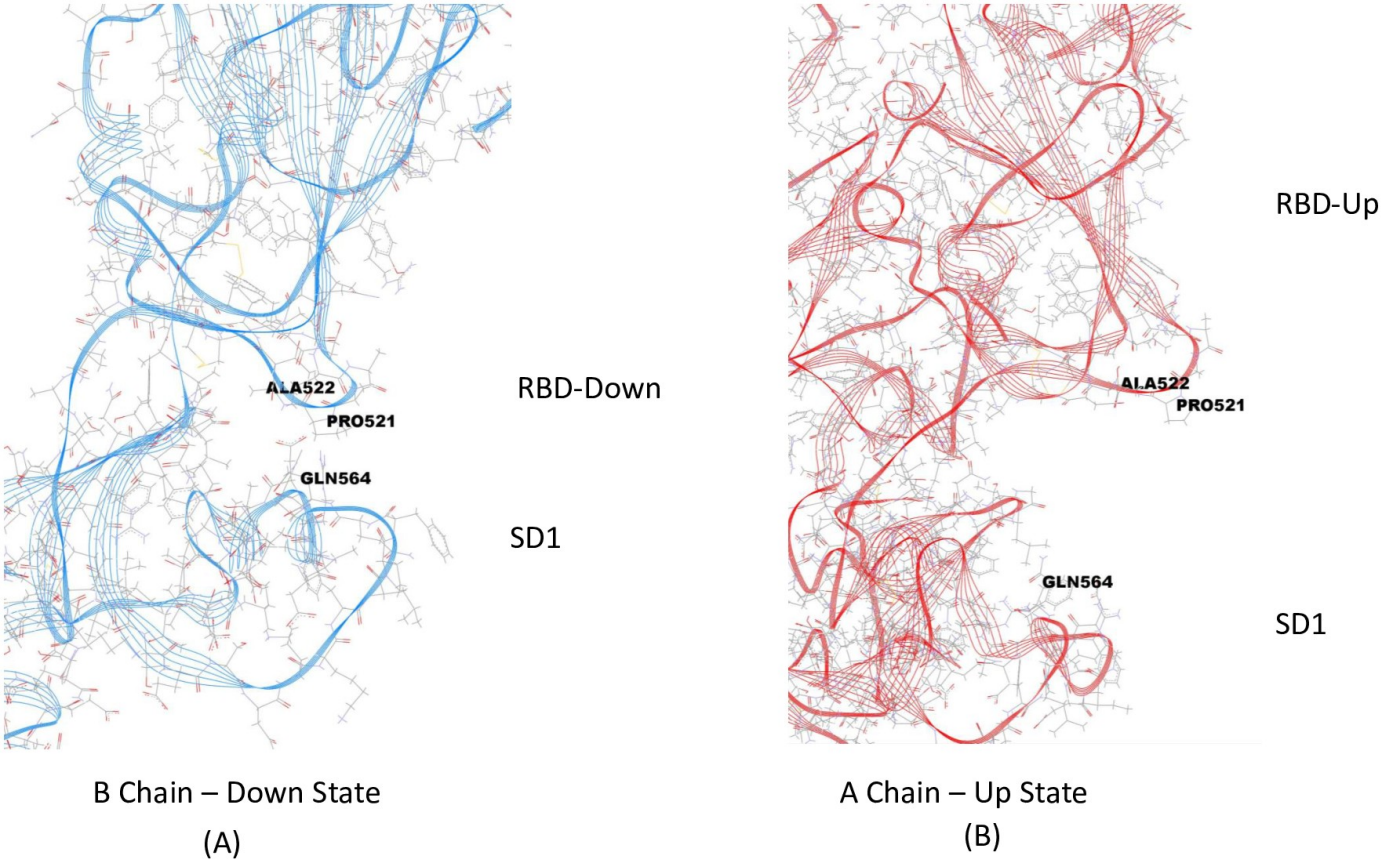
(A) Illustration of the area of dominant energetic interactions between SD1 and RBD of of Chain B Down: GLN564 with ALA520-PRO521-ALA522. (B) On right, the same SD1 area (GLN564) separated from the RBD residues ALA520-PRO521-ALA522.

**Fig 8:**
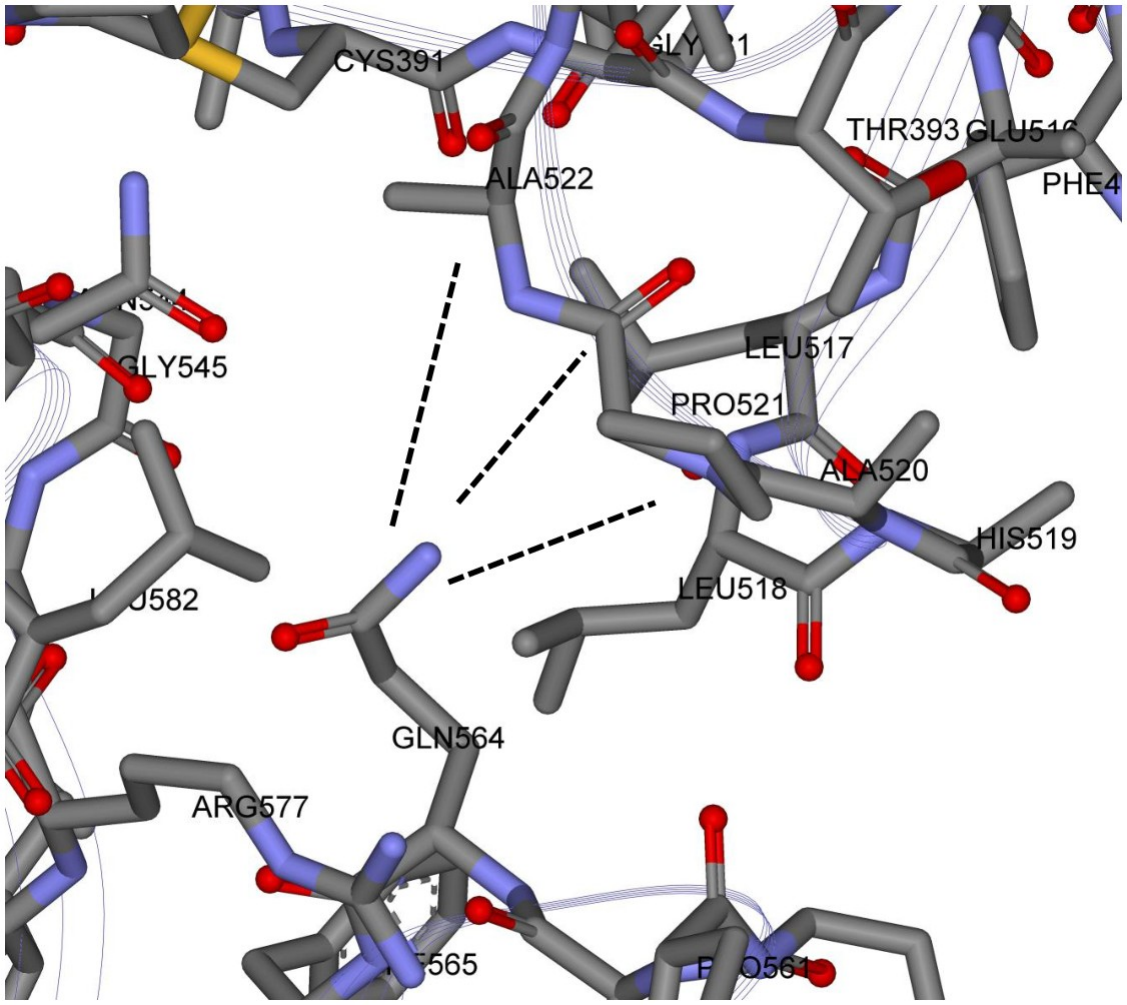
Zoomed View of possible molecular latch between the RBD and SD1 domain: Backbone atoms of ALA520 PRO 521 ALA 522 with the R-group of GLN 564. See S8 Table in S1 File for a complete listing of the dominant energetic interactions and their specific values.

**Fig 9:**
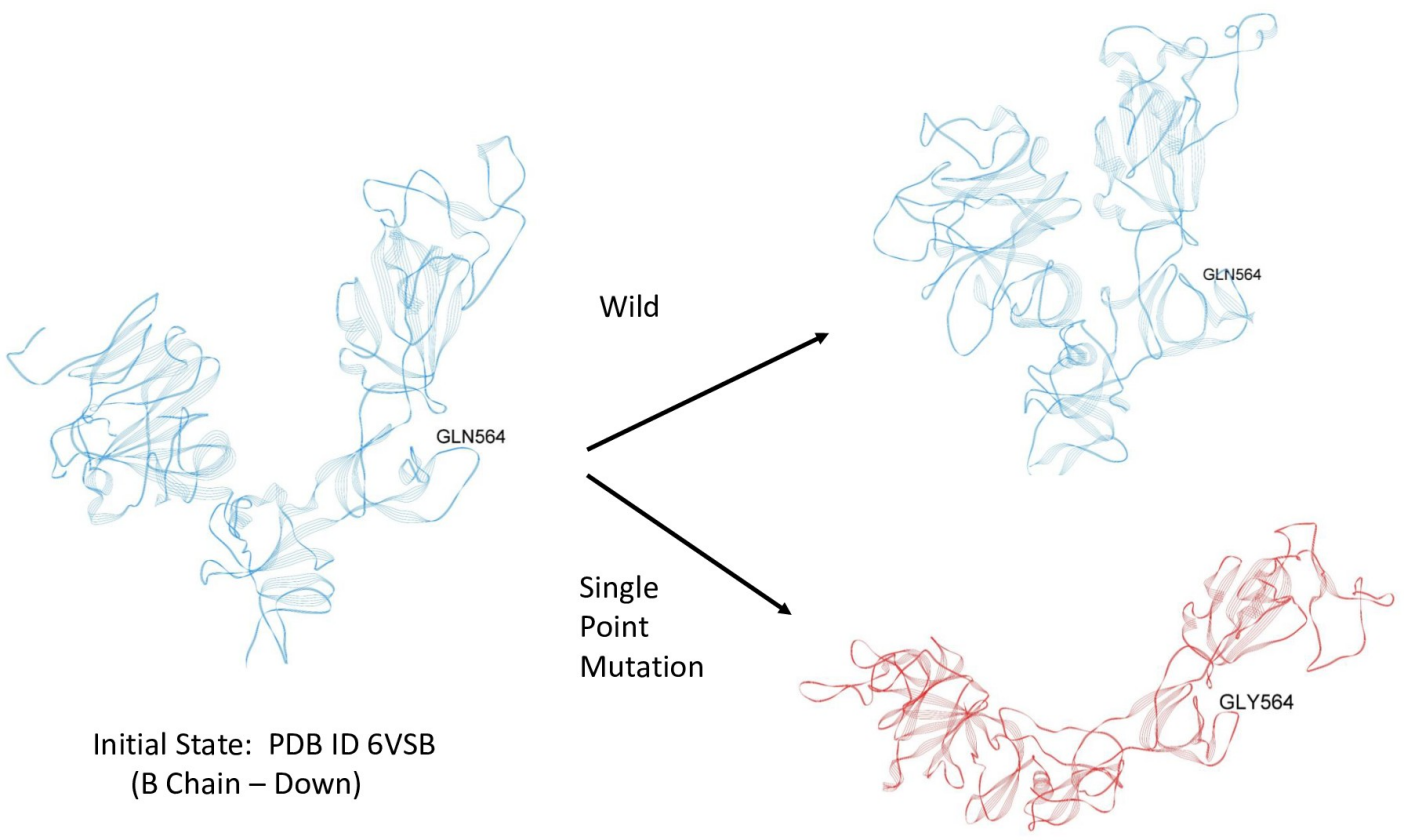
Initial dynamic test of possible latch over a period of approximately 0.1 *μ* sec. Blue—Wild; Red-Mutant.

For the Down state protomer, we observe conspicuous, strong partial atomic charge and van der Waals interactions between the R group of GLN564 in the SD1 domain with backbone atoms of the three sequential residues ALA520-PRO521-ALA522 in the RBD domain that were also seen in the cross chain glue point interactions. These specific atom-atom interactions are shown in more detail in Figs 7 and 8. Note the PRO521 interactions are dominated by van der Waals interactions in both instances (see S7 and S8 Tables in S1 File). It is interesting that these three residues play a dominant role in both interchain and intrachain interactions for the Down state protomer. The same key residues were also found with structure file 6VYB, as shown in S3 Fig in S1 File (cf. Fig 9)(see complete data listing in S9 Table in S1 File).

### Initial dynamic test of latch

**Fig 10:**
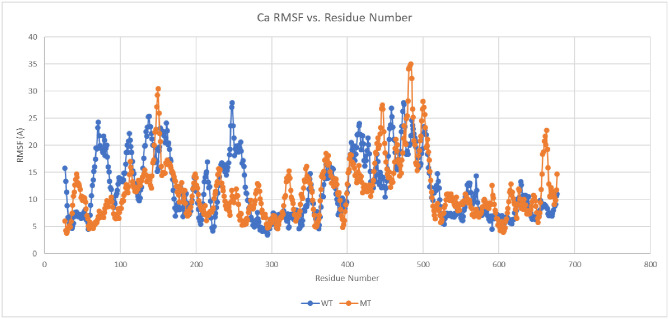
C-alpha (Ca) RMSF values for the S1 segment of the Spike protein over a period of approximately 0.1 *μ* sec. demonstrating the great degree of flexibility of the Spike protein. Blue—Wild; Orange-Mutant. NTD residues 21-318; RBD residues 319-527: SD1/SD2 residues 533-677.

In order to determine if these strong, but few residue interactions could possibly function as a molecular latch for the Down state protomer, we conducted some preliminary all-atom molecular dynamics simulation using NAMD2 over a total period of 0.1 *μ* sec. for both a wild S1 chain and a single point mutant S1 chain, both in the same initial down protomer state from PDB ID 6VSB. Thus, we use MD to probe the local dynamic stability beginning with the folded or minimum free energy state of the protein. For the mutant (single point mutation), we eliminated the R group glue point atoms of GLN 564 by replacing it with GLY 564, which is deemed one of the essential residues for the latch system, and did not alter in any way the three residue sequence ALA520-PRO521-ALA522 in the RBD domain or any other residues throughout the S1 chain. This mutation then effectively removes the R-group interaction of the backbone atoms of ALA520-PRO521-ALA522 thereby unhooking the latch. Note that ALA screening may not be effective at unhooking the latch in this case, since the R-group of ALA would still interact with its partner backbone atoms. Our trajectory analysis revealed the early release of the RBD from SD1 (within a few nanoseconds) and overall hinge opening and distal release of the RBD for this single point mutant, whereas the hinge angle and RBD relative positions were preserved in the wild state (Fig 9). (S1 Video in S1 File). For completeness, we also determined the C-alpha RMSF values for both wild and mutant (Fig 10) (S10 Table in S1 File). that clearly showed the relatively high degree of flexibility for the S1 Domain (RMSF values from 5 to 25 Å). The great degree of flexibility of the RBD is to be noted including the greatest degree (residues 480-500) that are associated with specific ACE2 binding domain residues [3].

Next, in order to partially test the importance of interchain interactions, we repeated the same mutation GLN564GLY across all protomers (one Up and two Down) of the complete trimeric Spike protein for a total period of approximately 1.0 microseconds. The results are shown in the supplemental movie S2 Video in S1 File. that demonstrate no changes in the Up or Down states of the RBD despite the quick hinge opening in the isolated protomer. Thus, even with a radical mutation that quickly unlatches an isolated Down state protomer, neighboring chain interactions stabilize the trimeric structure and help retain its specific conformational states.

## Conclusions

The all-atom energetic mappings are shown to be useful in attempting to understand the overall functionality of the Spike protein of the novel corona virus. The S2, fusion machinery domain, for example, also acts as a rigid base for the more flexible S1, prefusion domain, where flexibility is an important part of its function. The RBD is tethered to the SD1/SD2 domain by two peptide strings: one from the NTD (approximately residues 327-335) and the other connected to the SD1 domains (approximately residues 527-535). These peptide “strings” are stabilized or “tied together” by beta strand structures near the S1/S2 interface. The RBD domain in the Down state protomer is partially stabilized by the dominant energetic interactions of the backbone atoms of three of its residues (in sequence) with the R-group of a single residue from the SD1 domain, which we have called a possible latch. Ironically, these same three residues are involved in helping to stabilize the Up state protomer by interactions with the NTD of its Down state neighbor. This study raises a number of new questions about the origins of the Up and Down state protomers, including the reasons behind the occurrence of the Up state protomer, since as we have shown here, the Down state is partially stabilized by not only the potential latch, but also neighboring chain interactions between the RBD and it’s NTD neighbor. It seems reasonable, however, that the process of “Down to Up” is irreversible in the absence of deliberate outside interference. We further note that the possible three residue latch of the RBD Down state (ALA520 PRO521 ALA522) along with GLN654 of SD1 are conserved across reported variants for SARS-CoV-2 [1]. All latch residues are also present in the closely related virions: SARS-CoV and the bat coronavirus RatG13; the latter being 96% homologous to SARS-CoV-2 [1]. All-atom molecular dynamic studies with an in-silico single point mutation of GLN564GLY demonstrated the dramatic structural change of the S1 domain with hinge opening and distal release of the RBD. The same mutation, however, across the entire trimeric structure did not result in latch release demonstrating the critical importance of cross chain interactions in stabilizing the Spike protein. For example, this may explain the lack of appearance of more than one Up chain as this may significantly destabilize the trimeric state. In addition, these results may lead to targeting of the noncovalent cross chain interactions as a means of dismantling and possibly disabling of the prefusion Spike protein. We also observed the greatest degree of flexibility of the RBD in its ACE2 peptide binding region. The implications of that remain to be determined as well as further pinpointing of the cross chain interactions and Spike protein stability including those across other human coronaviruses.

## Supporting iformation

S1 File (ZIP)

## References

[1] Wrapp D, Wang N, Corbett KS, Goldsmith JA, Hsieh CL, Abiona O, et al Cryo-EM structure of the 2019-nCoV spike in the prefusion conformation. Science. 2020;367(6483):1260–1263. 10.1126/science.abb2507 32075877 PMC7164637

[2] Walls AC, Park YJ, Tortorici MA, Wall A, McGuire AT, Veesler D. Structure, function and antigenicity of the SARS-CoV-2 spike glycoprotein. Biochemistry; 2020 Available from: http://biorxiv.org/lookup/doi/10.1101/2020.02.19.956581. 10.1016/j.cell.2020.02.058 32155444 PMC7102599

[3] Yan R, Zhang Y, Li Y, Xia L, Guo Y, Zhou Q. Structural basis for the recognition of SARS-CoV-2 by full-length human ACE2. Science. 2020;367(6485):1444 10.1126/science.abb2762 32132184 PMC7164635

[4] Henderson R, Edwards RJ, Mansouri K, Janowska K, Stalls V, Gobeil SMC, et al Controlling the SARS-CoV-2 spike glycoprotein conformation. Nature Structural & Molecular Biology. 2020;. 10.1038/s41594-020-0479-4 PMC8581954 32699321

[5] Walls AC, Xiong X, Park YJ, Tortorici MA, Snijder J, Quispe J, et al Unexpected Receptor Functional Mimicry Elucidates Activation of Coronavirus Fusion. Cell. 2019;176(5):1026–1039.e15. 10.1016/j.cell.2018.12.028 30712865 PMC6751136

[6] Li T, Zheng Q, Yu H, Wu D, Xue W, Zhang Y, et al Characterization of the SARS-CoV-2 Spike in an Early Prefusion Conformation. Molecular Biology; 2020 Available from: http://biorxiv.org/lookup/doi/10.1101/2020.03.16.994152.

[7] Shaw DE. Molecular Dynamics Simulations Related to SARS-CoV-2; 2020 Available from: http://www.deshawresearch.com/resources_sarscov2.html.

[8] Cai Y, Zhang J, Xiao T, Peng H, Sterling SM, Walsh RM, et al Distinct conformational states of SARS-CoV-2 spike protein. Science. 2020; p. eabd4251 10.1126/science.abd4251 PMC7464562 32694201

[9] McCallum M, Walls AC, Bowen JE, Corti D, Veesler D. Structure-guided covalent stabilization of coronavirus spike glycoprotein trimers in the closed conformation. Nature Structural & Molecular Biology. 2020;. 10.1038/s41594-020-0483-8 32753755 PMC7541350

[10] Bastidas OH, Green B, Sprague M, Peters MH. Few Ramachandran Angle Changes Provide Interaction Strength Increase in A*β*42 versus A*β*40 Amyloid Fibrils. Scientific Reports. 2016;6(1):36499 10.1038/srep36499 27808259 PMC5093553

[11] Krall A, Brunn J, Kankanala S, Peters MH. A simple contact mapping algorithm for identifying potential peptide mimetics in protein-protein interaction partners: Contact Mapping for Potential Peptide Mimetics. Proteins: Structure, Function, and Bioinformatics. 2014;82(9):2253–2262. 10.1002/prot.24592 PMC4369124 24756879

[12] Phillips JC, Braun R, Wang W, Gumbart J, Tajkhorshid E, Villa E, et al Scalable molecular dynamics with NAMD. Journal of Computational Chemistry. 2005;26(16):1781–1802. 10.1002/jcc.20289 16222654 PMC2486339

[13] Jo S, Kim T, Iyer VG, Im W. CHARMM-GUI: a web-based graphical user interface for CHARMM. Journal of Computational Chemistry. 2008;29(11):1859–1865. 10.1002/jcc.20945 18351591

