# Supplementary figures and images for: Static all-atom energetic mappings of the SARS-Cov-2 spike protein and dynamic stability analysis of “Up” versus “Down” protomer states
Source: PLoS One. 2020 Nov 10;15(11):e0241168. doi: 10.1371/journal.pone.0241168 (PMC7654774; doi:10.1371/journal.pone.0241168)

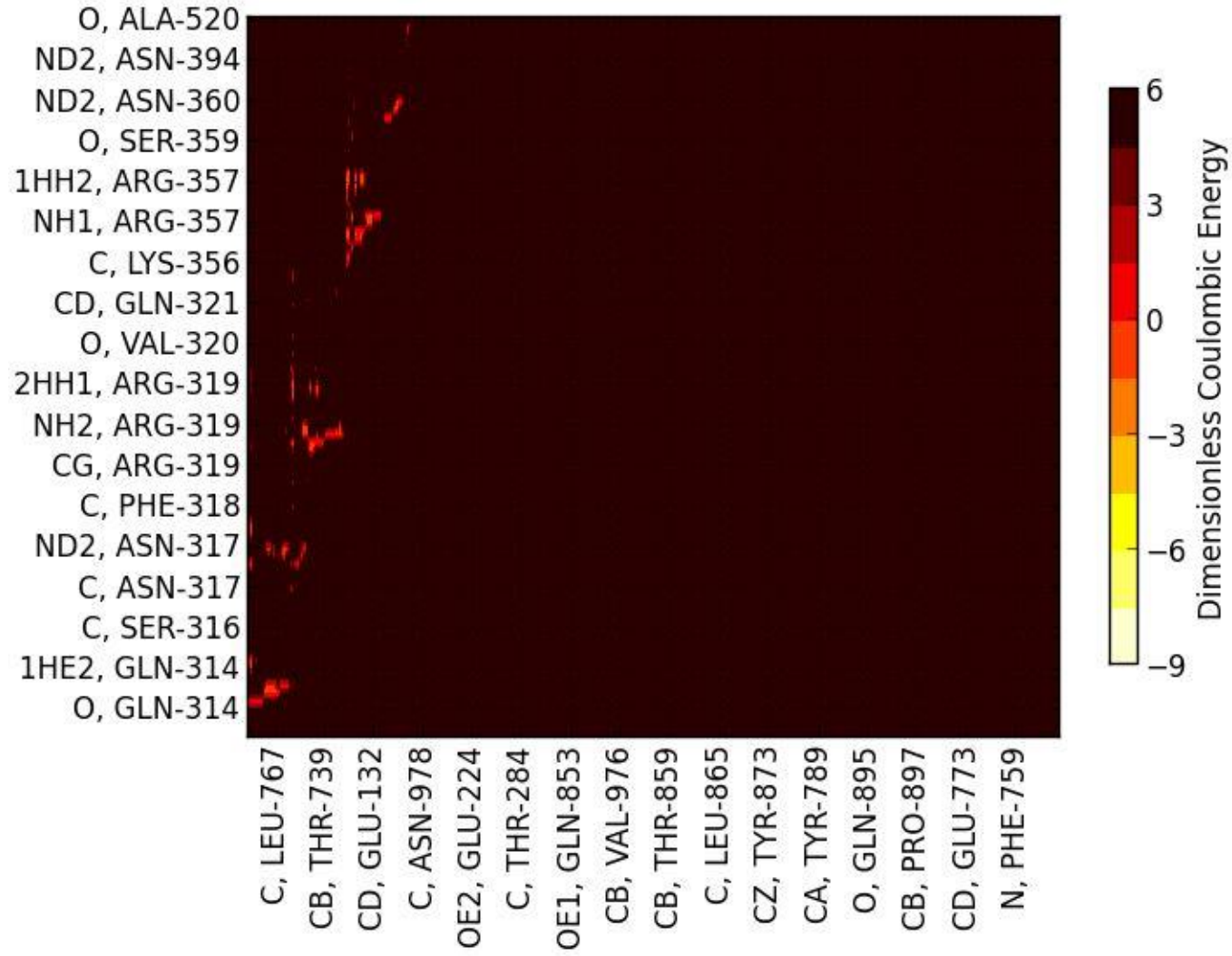

Supplement: S1 File — (ZIP) [file pone.0241168.s001.zip › Final Supplementary/S1_Fig.pdf]

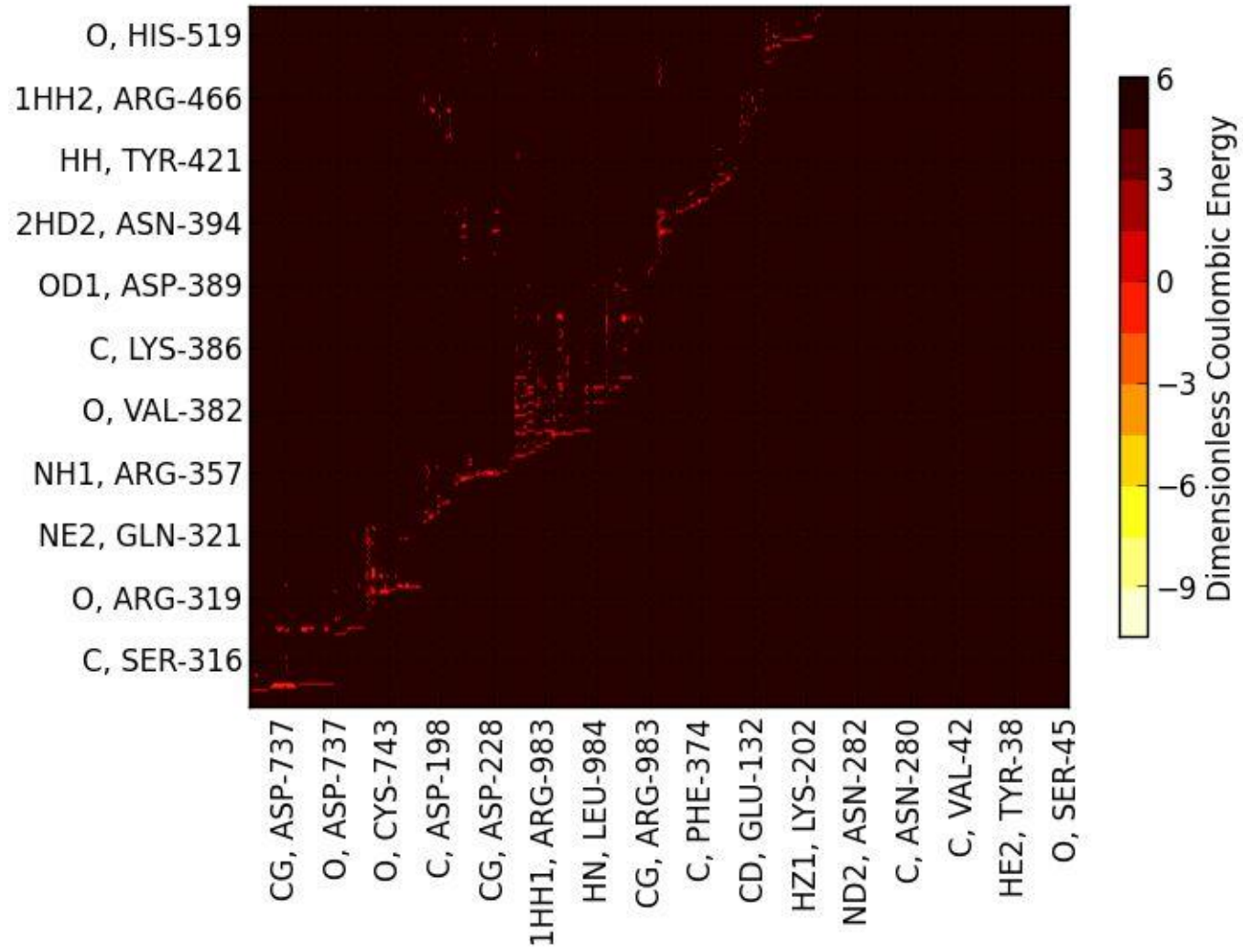

Supplement: S1 File — (ZIP) [file pone.0241168.s001.zip › Final Supplementary/S2_Fig.pdf]

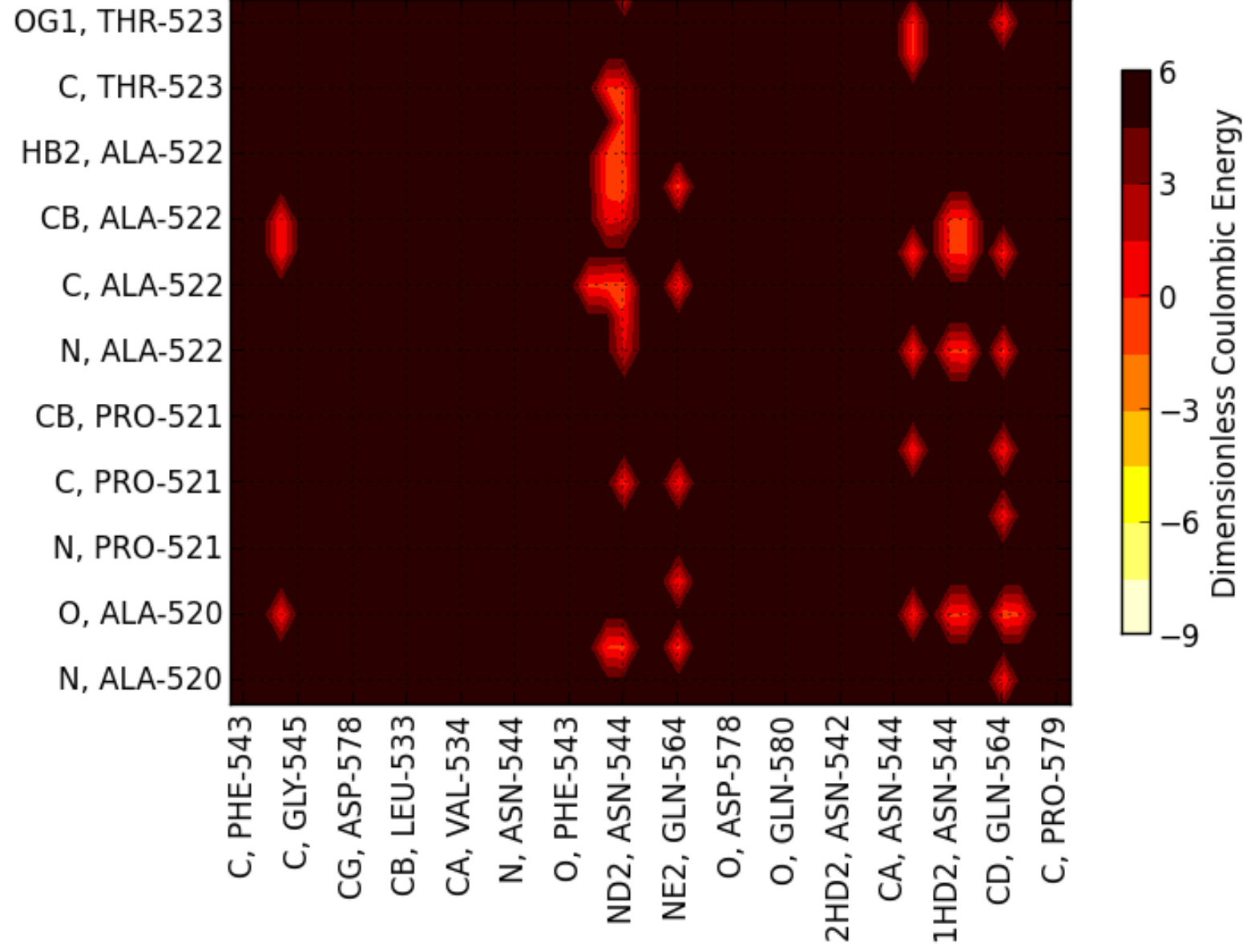

Supplement: S1 File — (ZIP) [file pone.0241168.s001.zip › Final Supplementary/S3_Fig.pdf]
